# Preliminary Stages for COVID-19 Detection Using Image Processing

**DOI:** 10.3390/diagnostics12123171

**Published:** 2022-12-15

**Authors:** Taqwa Ahmed Alhaj, Inshirah Idris, Fatin A. Elhaj, Tusneem A. Elhassan, Muhammad Akmal Remli, Maheyzah Md Siraj, Mohd Shafry Mohd Rahim

**Affiliations:** 1 School of Computing, Universiti Teknologi Malaysia, Johor Bahru 81310, Johor, Malaysia; 2 Institute For Artificial Intelligence & Big Data, Universiti Malaysia Kelantan, City Campus, Pengkalan Chepa, Kota Bharu 16100, Kelantan, Malaysia; 3 Department of Computer Science, University of Wad Medani Ahlia, Wad Madani 21111, Sudan; 4 College of Art, Science and Information Technology, University of Khorfakkan, Khorfakkan-Sharjah P.O. Box 18119, United Arab Emirates; 5 Faculty of Data Science and Computing, Universiti Malaysia Kelantan, City Campus, Pengkalan Chepa, Kota Bharu 16100, Kelantan, Malaysia

**Keywords:** COVID-19, preprocessing, augmentation, segmentation, feature extraction, transfer learning, X-ray, CT

## Abstract

COVID-19 was first discovered in December 2019 in Wuhan. There have been reports of thousands of illnesses and hundreds of deaths in almost every region of the world. Medical images, when combined with cutting-edge technology such as artificial intelligence, have the potential to improve the efficiency of the public health system and deliver faster and more reliable findings in the detection of COVID-19. The process of developing the COVID-19 diagnostic system begins with image accusation and proceeds via preprocessing, feature extraction, and classification. According to literature review, several attempts to develop taxonomies for COVID-19 detection using image processing methods have been introduced. However, most of these adhere to a standard category that exclusively considers classification methods. Therefore, in this study a new taxonomy for the early stages of COVID-19 detection is proposed. It attempts to offer a full grasp of image processing in COVID-19 while considering all phases required prior to classification. The survey concludes with a discussion of outstanding concerns and future directions.

## 1. Introduction

Throughout history mankind has experienced pandemics, of which several have been extremely disastrous [1]. Over the past century, the COVID-19 pandemic is considered the deadliest, even worse than the Spanish flu [2]. Beginning December 2019, the rapid spread of COVID-19 has caused widespread concern throughout the world. Hundreds of deaths and thousands of illnesses have been reported in practically every part of the world [1]. Therefore, early detection of COVID-19 is critical for limiting the virus from spreading and to providing care to prevent complications. One of the most important diagnostic tools for identifying and distinguishing infections in humans is reverse transcription-polymerase chain reaction (RT-PCR).

X-ray images and computed tomography scans (CT scans) are additional diagnostic tools used for identifying COVID-19 [3]. The most common radiological findings in COVID-19 patients are bilateral and multifocal ground-glass opacities and consolidations, especially in the basal and peripheral sites. Based on CT or X-ray of the images of the lungs, doctors can observe and examine the signs associated with the COVID-19 deformations. However, when examining the results of these imaging techniques, radiologists may experience technical problems that lead to decreased sensitivity [4]. Therefore, utilizing modern technologies to combat COVID-19 can increase the performance of the public health system [5]. Such supplementary technologies include artificial intelligence (AI), which can be used to combat this virus through population screening, alerts, recommendations for infection control, learning-prediction models, improved drug discovery, treatment design, and outlining follow-ups for COVID-19 patients [3]. Additionally, AI can help in the diagnosis of a variety of diseases, such as brain tumors from MR images, various types of brain disorders from EEG, breast cancer from mammographic images, and pulmonary diseases such as COVID-19 from ultrasound, X-rays, and CT-Scas [6]. These approaches allow for the evaluation of specific segment regions and the acquisition of precise structures in chest images for diagnostic purposes [7].

The development of the COVID-19 diagnostic system begins with image acquisition and continues through the preprocessing, feature extraction, and classification phases [8]. Therefore, significant work must be committed to improving the preliminary phases of these systems in order to improve the accuracy of COVID-19 diagnostic systems, including preprocessing, augmentation, segmentation, and feature extraction, which is the subject of this work. Preprocessing involves the removal of undesirable artifacts and distribution associated with the image to obtain useful features [9]. While segmentation is the process of identifying the region of interest (ROI) in an image to gather relevant information that is necessary to enhance subsequent classification or object recognition tasks [10,11], augmentation is the process of producing synthetic images using many transformation techniques. In this phase, it is crucial to address the issues of insufficient data and unbalanced distribution [12]. Meanwhile, feature extraction is the process of learning significant image representation while preserving original information [13].

All the components mentioned above are further discussed in detail in the following sections. Specifically, the contributions of our work is listed as follows:

1. We analyze how various preprocessing techniques can be used to enhance feature extraction in each of the investigated works.
2. We present a detailed discussion of the different segmentation approaches employed in each reviewed paper, with the goal of delivering significant features that are reliable for COVID-19 detection.
3. We provide a comprehensive analysis of the various augmentation methods employed to address the issue of a lack of images available for COVID-19 detection.
4. We present a complete investigation of the various feature extraction techniques used to distinguish COVID-19 images from normal images.

The study begins by introducing fundamental concepts related to COVID-19. Section 3 investigates many sources of information used by various authors in research on the detection of COVID-19. Section 4 then compares several related surveys. Subsequently, Section 5 introduces the proposed taxonomy of the earliest stages of COVID-19 detection. Finally, Section 10 summarizes the discussion and points out future research directions.

## 2. Concept and Background

The COVID-19 index case was discovered in Wuhan, Hubei Province, People’s Republic of China (PRC). The acute respiratory syndrome coronavirus 2, or COVID-19, was identified and categorized as an infectious virus (SARS CoV-2) [14]. According to investigations, COVID-19 most likely originated in Wuhan’s Huanan Seafood Market, and by December 2019, the PRC government had officially declared an additional 27 cases [15]. The COVID-19 virus epidemic began during the PRC’s spring carnival, when many people from all over the world traveled there. The massive influx of people from different countries all over the world acted as a catalyst for the spread of the virus both within China and across international borders to other countries [14]. The original SARS-CoV virus was contracted from a cat, and the MERS-CoV virus is acquired from a dromedary; therefore, COVID-19 is classified as a zoonotic disease because it is thought to have spread from animals to humans through bats [16].

Viral replication occurs in the lung cells after the virus enters them via the respiratory system. COVID-19 is extremely difficult to diagnose and cure due to the RNA that composes its mutational characteristics [6]. Furthermore, the rapid spread of COVID-19 is mainly due to airborne and physical contact, such as hand contact with an infected person [6]. According to comparative studies, men are more likely to contract the infection than women because they are more frequently exposed to it. Despite that, there have been no deaths reported for children between the ages of 0 and 9. In contrast to healthy subjects, COVID-19-induced pneumonia subjects suffer faster spread of respiratory problems [16]. Frequent symptoms as a result of viral infection are fever and cough. The more severe effects of the virus are highly associated with acute respiratory distress syndrome (ARDS), severe interstitial pneumonia, and subsequent multi-organ failure, which have high mortality rates [17]. As there is currently no cure for COVID-19, many infection-control measures have been implemented. However, previous attempts in past years to deal with MERS-CoV have resulted in considerable improvements in hospital infection control practices. Multiple countries have used non-pharmaceutical interventions (NPIs) to inhibit the spread of the virus [18]. Simultaneously, several vaccinations and anti-virals have been researched and prioritized by scientists globally. Multiple vaccine programs have been effective in clinical trials using recombinant DNA, mRNA, live attenuated virus, S-protein subunits, virus-like particles, and viral vectors across multiple vaccine initiatives [19]. Most of these efforts were inherited from SARS-CoV and MERS-related to create a vaccine against the novel coronaviruses. SARS-CoV-2 utilizes the same receptor as SARS-CoV on the host cell, specifically, human Angiotensin Converting Enzyme 2 (hACE2), and shares around 79% genetic similarity with SARS-CoV [2].

## 3. Source of Information

The use of COVID-19 computer vision diagnostic tools from a number of imaging modalities, including X-ray, ultrasound, and CT, can provide doctors with an automated second reading, facilitating the diagnosis of COVID-19 patients [20]. Because image classification techniques are regarded as a low-cost and accurate diagnostic tool, a number of datasets have been created to aid research in the field of COVID-19 diagnosis. These datasets include images from X-rays, CT scans, and ultrasounds. The most common type of published dataset are X-ray datasets, followed by CT scans, then ultrasounds. The following sections discuss the most commonly used dataset types.

### 3.1. X-ray

X-rays are a form of electromagnetic wave radiation. They penetrate the body to generate a two-dimensional image of the inside of the human body. X-ray images portray various body parts in black and white. This occurs due to the differences in various tissues with regard to absorbing different amounts of radiation. Therefore, bones appear as white because the calcium in bones absorbs most X-rays. In addition, the color of hard films appears grey because of the diminished light absorption of fat and other soft tissues. In contrast, the lungs appear black because of the very X-ray low absorption of air [21]. X-ray imaging is a low-cost method for detecting lung infections, and can be used to diagnose COVID-19. In X-ray images of COVID-19 patients, patchy infiltrates or opacities that resemble other viral pneumonia symptoms are commonly observed. Usually, there are no abnormalities shown in X-ray images during the early stages of COVID-19; however, the symptoms gradually appear as a characteristic unilateral patchy infiltration at the mid-zone and upper or lower zone of the lungs, with indications of consolidation on occasion [20]. Despite this, there are many limitations of X-ray datasets, such as the limited number of available X-ray scans labeled as positive COVID-19 infections. Furthermore, no unified data, classes, or evaluation protocols have been presented. Regardless, numerous X-ray datasets have been published to improve COVID-19 detection techniques. Table 1 lists and briefly describes a number of COVID-19 related X-ray datasets.

### 3.2. Computed Tomography (CT)

CT was the first non-invasive radiological approach to allow for the creation of tomographic images of all parts of the human body without superposition of nearby structures [22]. CT can be used to scan the body by using X-rays to obtain comprehensive cross-sectional images. These various images are then combined to create 3D images. During CT scanning, the patient lies on a table. Slowly, the table moves across the center of a gigantic X-ray machine. CT scans can provide images of every parts of human anatomy, including organs, bones, and blood vessels, which are in turn used by doctors to assist in diagnosing and managing a wide range of medical issues [21]. CT scanning is the most widely recommended screening tool for early COVID-19 detection, as it is a highly viable technology for this purpose. However, there are obvious practical disadvantages to CT, such as need for the patient to be exposed to excessive radiation, high cost, availability of advanced equipment, the necessity for extensive sterilizing, and limitations with respect to patient mobility [23]. CT scan datasets have been primarily used to segment specific thoracic regions in order to diagnose COVID-19 patients. Table 2 summarizes relevant CT image datasets.

### 3.3. Ultrasound

Ultrasound, often known as sonography, is a type of imaging. Ultrasonic instruments are commonly employed by healthcare professionals. The use of ultrasound imaging in medical diagnosis is widely established due to its noninvasive nature, low cost, capacity to produce real-time images, and ongoing improvement in image quality. It examines internal bodily organs and structures using high-frequency sound waves [21]. Unlike X-rays, ultrasound does not expose patients to radiation. The patient lies on a table for an ultrasound test. Meanwhile, a transducer is moved across the body by a professional technician or doctor. Sound waves are emitted by the transducer and bounce off the tissues inside the body. The waves that bounce back are likewise recorded by the transducer. The ultrasound equipment generates images based on the sound waves [21]. Depending on how the ultrasonic scanner is configured, it can produce real-time tomographic images of ultrasound scattering, real-time images of blood and tissue mobility, elasticity, and tissue flow (perfusion). All these images are constructed line by line by delivering ultrasonic pulses into the tissue and capturing the reflected radiofrequency signals. When an infection occurs in its early stages, ultrasound can detect pleural and interstitial thickening, subpleural consolidation, and other physiological events associated with changes in the lung structure. According to studies, the major criteria enabling COVID-19 detection are anomalies in bilateral B-lines and recognizable lesions in the bilateral lower lobes [23]. There are various limitations on ultrasound imaging that restrict its effectiveness for lesional detection and as a guiding technique, including:

1. Image acquisition is user dependent.
2. The field of view is limited.
3. Ultrasound images are typically acquired off-plane compared to the true axial, sagittal, or coronal planes, resulting in difficulty in correlating them with other cross-sectional imaging methods.
4. Lesional identification can be difficult due to its echogenicity relative to the organ that is scrutinized.
5. The quality of imaging can be affected by the physical characteristics of the patient [24].

Regardless of their benefits, ultrasound datasets are rarely used. Table 3 provides descriptions of datasets from the literature.

## 4. Related Surveys

The use of DL for COVID-19 detection has been covered in a number of studies, either exclusively, such as in [25,26,27,28], or implicitly, such as in [6,14,29,30].

Shoeibi et al. [6] used DL networks to conduct a comprehensive review of completed COVID-19 diagnosis studies. This study discussed the public datasets that can be used to diagnose and predict COVID-19. In addition, the authors provided the most advanced DL approaches used for COVID-19 diagnosis, segmentation, and forecasting. However, their discussion of the datasets used was brief and superficial. Aside from not being exhaustive, the DL algorithms for the detection stage have been covered in a number of other related works, and the description of the segmentation phase is relatively brief.

Bhattacharya et al. [14] summarized the most recent research on DL applications for COVID-19 medical image processing. The authors present an outline of DL and its applications to healthcare that have been discovered in the recent decade. Following that, they describe many of the obstacles and issues associated with DL implementations for COVID-19 medical image processing. However, there is no comprehensive discussion of the outlined state-of-the-art of the stages prior to detection. Moreover, the paper does not include a comparative discussion of other closely related surveys.

Alghamdi et al. [26] presented a comprehensive review of the diverse DL methods used to detect COVID-19 via X-ray images and CT scans. Additionally, the most prevalent pretrained CNN architectures were described. However, when compared to the DL architecture modeling with the methodologies used to explain classification decisions, which is provided in a separate section, their classification criterion lacks clarity and interpretability. Furthermore, most of the papers examined concentrate solely on transfer learning methodologies.

Chen et al. [29] investigated AI-based imaging analysis methods for COVID-19 as well as chest imaging analysis of two common viral pneumonias that can serve as a reference for COVID-19 analysis. In addition, methods for AI-assisted CXR imaging analysis for COVID-19 were discussed. However, their survey categorization is complicated by its combining of the image processing, image segmentation, and image extraction stages into one section. Aside from the fact that it is not exhaustive, the segmentation and extraction processes are duplicated in another section, which appears repetitive and inconsistent.

Aishwarya et al. [31] investigated various COVID-19 detection techniques based on ML and DL that can assist clinicians and doctors in swiftly identifying COVID-19 cases. The authors reviewed several DL techniques, including 3D and 2D analysis of chest CT images. Meanwhile, thet examined ML approaches using models such as RF, ARIMA, SVR, CUBIST, and Gradient Boosting to make precise predictions. However, their survey focused on the architecture of DL, which appears to have been replicated and addressed in other survey studies, with no meaningful insights from the survey discussed.

Sufian et al. [27] tried to bring potentialities and challenges of deep transfer learning, edge computing and their related issues to the topic if mitigating the COVID-19 pandemic. They proposed a conceptual combined model and presented its scope and the future challenges of working at critical sites and real data. However, the main aim of the study was limited to DL implementation. This work does not specifically address the initial phases prior to detection. Specifically, their investigation of related articles was insufficient.

Khan et al. [30] presented a comprehensive review of DL techniques based on image and region-level analysis of COVID-19 infection. The taxonomy of the survey study demonstrates the efficacy of classification, segmentation, and multi-stage techniques for detecting and diagnosing COVID-19 infection from radiological images. They provide an overview of each study by detailing the dataset, the number of classes, partitioning, model structure, and the performance evaluation criteria. Nonetheless, there is no explanation of the other aspects of the proposed taxonomy, such as the preprocessing phase. Furthermore, several of their classification criteria may require clarification.

Subramanian et al. [28] investigated the existing DL methods for detecting COVID-19 from lung images by summarizing the datasets used by each method. They classified the material into three categories: transfer learning and fine-tuning, innovative architecture, and alternative techniques. The approaches used for each category are listed as well. Finally, the challenges of using DL methods for COVID-19 detection are discussed, as well as potential future trends in this research area. Most of their discussion of the surveyed publications is limited to datasets. Furthermore, COVID-19 detection phases such as preprocessing, segmentation, and augmentation are only briefly studied, and in a relatively limited area.

Shyni and Chitra [32] assessed recent DL techniques for COVID-19 diagnosis, emphasizing the significance of preprocessing medical images, transfer learning, and data augmentation techniques to address data scarcity issues. Furthermore, the use of pre-trained models to reduce time was summarized, as well as the importance of medical imaging in the automatic detection of COVID-19. This article discusses the potential of developing highly effective CNN models using medical images for disease detection. However, these phases are only considered in a limited way, and the state-of-the-art in each phase is not mentioned. Furthermore, the survey is focused on DL architecture, which appears to have been replicated and addressed in other survey research.

In contrast, our work carefully investigates each stage from a variety of aspects, including image preprocessing, augmentation, segmentation, and feature extraction. Additionally, we look at different information sources pertinent to needs and difficulties. Modern pre-trained algorithms that extract key features using transfer learning are examined as well. The following Table 4 compares current surveys of COVID-19 detection approaches to our study in key areas.

## 5. Taxonomy of the Preliminary Stages for COVID-19 Detection

**Figure 1:**
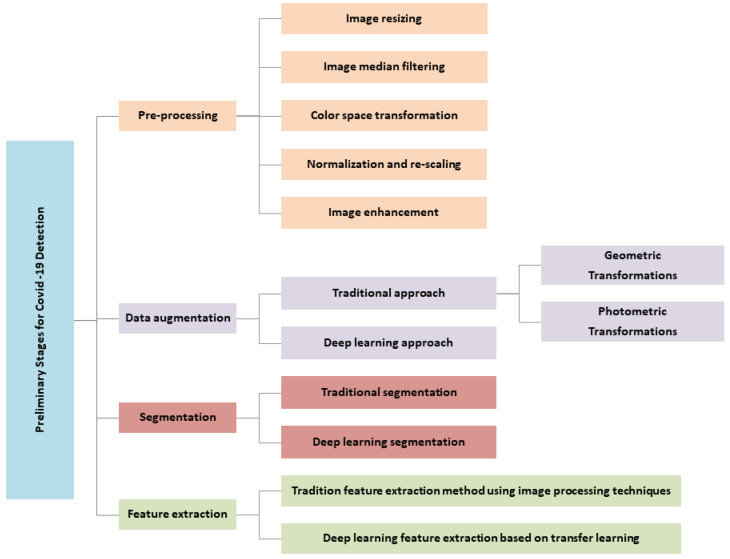
Proposed taxonomy of the preliminary stages of COVID-19 detection.

A thorough analysis of the literature reveals several attempts to develop taxonomies for COVID-19 detection using image processing techniques. Most of them use a categorization criterion that is solely based on classification techniques. In this study, a brand-new taxonomy for the early stages of COVID-19 detection is suggested, as shown in Figure 1. It strives to present a thorough understanding of image processing in COVID-19 by considering all the stages required prior to the classification process. These early stages are intended to provide strategic guidance on how to achieve high classification performance for accurate COVID-19 detection. These preliminary stages can be broken down into four categories: preprocessing, image augmentation, image segmentation, and feature extraction. The breadth of all these aspects is described in the following sections.

## 6. Preprocessing

Preprocessing stage is a key step for obtaining meaningful information in image detection and classification [34]. Most preprocessing methods are used for such common purposes as:

- Reducing or eliminating the impact of data variability on model performance, as images are obtained from a variety of datasets with varied image sizes and acquisition conditions [35].
- Improving the contrast of an image [12].
- Producing accurate and consistent findings when classifying COVID-19 from chest images.
- Making the illness zone in the image more evident in comparison to the original image [12].

According to the literature, the preprocessing stage includes many operations. The following section provides a full explanation of each of them.

### 6.1. Image Resizing

Images must be resized and scaled because they contain many letters, medical symbols, and art craft, and as they come from diverse sources with varying sizes [36]. In Ismael et al. [37], input chest X-ray images were first scaled to 224 × 224 pixels for compatibility with CNN models. Furthermore, many other researchers [35,38,39,40,41,42,43,44,45,46,47,48,49] have resized CT and X-ray images to the same 224 × 224 size. Meanwhile, other researchers, such as [50,51,52,53], have reduced all the images to 512 × 512 pixels in size. In addition, Jain et al. [54] observed the images in the dataset to determine the minimum height and width. After discovering the minimum dimension, all the dataset images can be shrunk to this size.The minimum dimension obtained in their research was 640 × 640 pixels. Similarly, the authors of [55] trained their proposed model using a dataset consisting of 200 COVID-19 X-rays, 250 viral pneumonia X-rays, and 250 normal X-rays, all of which were shrunk to 200 × 200 pixels in height and width. Various further resizings have been carried out, including 60 × 60, 299 × 299, and 256 × 256 by [36,56,57], respectively.

### 6.2. Image Filtering

Filtering techniques preserve important information in an image while filtering out any noise. Median filtering is a nonlinear filtering approach, and is commonly employed in digital image processing due to its excellent edge keeping properties and ability to reduce impulse noise [58]. For example, Rajaraman et al. applied median filtering for noise removal and edge preservation to preprocess lung images via cropping [59]. Furthermore, Jamil et al. were able to eliminate any visible noise from X-ray images using 2D Gaussian filtering [60], while Arias et al. [38] reduced unnecessary information from X-ray images for lung segmentation by filtering the images according to frontal and lateral image projections. A modified anisotropic diffusion filtering (MADF) approach was applied in [34] to preserve delicate information while minimizing noise and distortion in the image.

### 6.3. Color Space Transformation

In the field of computer vision, color-based transformation methods are commonly used for image enhancement [12]. In [61,62], the authors shown that preprocessing using fuzzy color image enhancement technique could increase classification performance greatly. Ahsan et al. [34] converted X-ray images from RGB to grayscale and identified the region of interest (ROI) by removing the unwanted regions. According to [62], data from X-ray images can be reconstructed using the fuzzy color technique, followed by stacking of the images in a structure with the original images. The fuzzy color method works by dividing the supplied data into blurred windows. Each pixel in the image has a degree of membership with respect to each window, which is determined based on the distance between the pixel and the window. The membership degrees are used to calculate image variance. In this stage, the weights of the images of each blurred window are added together, and the output image is produced using the average. The degree of membership is used to indicate the weight value of each pixel. The color conversion method is applied to convert RGB images to grayscale. In [56], all CT images of patients were converted to grayscale.

### 6.4. Normalization and Rescaling

Normalization of data is an important step in the preprocessing stage, and is commonly employed in DL models to preserve numerical stability. For example, a CNN model can be trained faster and its gradient descent is more stable if the data are normalized [41]. ImageNet statistics have been used by several authors, including [53,54]. In Jain et al. [54], each pixel of three channels in an image was normalized. For example, channel 1 has a mean of 0.485 and a standard deviation of 0.229, channel 2 has a mean of 0.456 and a standard deviation of 0.224, and channel 3 has a mean of 0.406 and a standard deviation of 0.225. Other researchers, such as [41,42], have rescaled the pixel value of the image to the interval [0, 1]. In [51], the authors used min-max scaling to normalize the pixels to [0, 1], then subtracted 0.5 from each pixel. As a result, the pixel values were adjusted to [0.5, 0.5]. Furthermore, [47,52,63] standardized all pixel values to a range of [0, 1] based on min–max feature scaling.

### 6.5. Image Enhancement

In [64], contrast enhancement was conducted separately on each image in the original dataset using the image contrast enhancement method throughout the process of constructing the enhancement dataset. The noise in the original dataset was wholly eliminated in this manner, resulting in the best contrast. The image contrast enhancement technique was first developed as a solution for unclear X-ray images [64].

## 7. Data Augmentation

To achieve consistent results, classification models require a significant amount of data, as they have been shown to perform better on larger datasets. On the other hand, there are very few training samples available in medical imaging datasets, and obtaining substantial amounts of medical data is challenging. This is a major concern when utilizing DL algorithms to interpret medical images, as it can be very costly and time-consuming [65]. Therefore, data augmentation is used to address these issues [65]. Data augmentation is a technique for increasing the number of samples by applying a series of transformations [12] while maintaining class labels. Augmentation increases image variability [41] and helps to avoid over-fitting [65]. Data augmentation can further enhance the performance of training models, thereby producing more impactful results [66]. There are two categories of data augmentation, namely, the traditional and DL approaches.

### 7.1. Traditional Data Augmentation Approach

Traditional data augmentation methods include geometric transformations and photometric transformations.

#### 7.1.1. Geometric Transformations

Several geometric transformation techniques have been used to improve DL models in recent studies of COVID-19 detection from images. Examples of these techniques include flipping, cropping, rotation, translation, zooming, shifting, scaling, and noise injection. While most researchers use two or more techniques, others use only one technique. For instance, in [67], flipping, zooming, and width shifting were used as data augmentation techniques to reduce the bias caused by the properties of CXR images. Joshi et al. [66] used image scaling and rotation to increase the original dataset CXR images by five. Their findings indicate that augmentation leads to improved performance. Chowdhury et al. [68] presented a collection of three recently published public X-ray datasets and achieved 98% accuracy using rotation, scaling, and translation.

Ibrahim et al. [42] used flipping, rotating, and skewing to increase the number of training images at two points, first in dataset preparation and then in data preprocessing, in order to attain efficient and reliable accuracy.Augmentations were applied twice to increase dataset size, which in turn has an impact on system accuracy.

Ahuja et al. [69] proposed a novel data augmentation method on a CT image dataset based on three levels of stationary wavelet transformation to solve overfitting problems. Subsequently, images were randomly rotated, sheared, and translated. Their results showed that this model’s accuracy outperforms alternatives even in the absence of the novel data augmentation method.

Yoo et al. [70] used a variety of data augmentation techniques to improve CNN model training and classification accuracy using a variety of chest X-ray datasets. The images were rotated, translated, and flipped horizontally. In certain cases, translation and rotation were used simultaneously. Similarly, Abbas et al. [71] employed the same techniques (flipping, rotating, and translating). In [65], the authors used different augmentation methods to prevent over-fitting in order to classify COVID-19-infected patients using chest X-rays. These methods included rotation, zooming, and image sharing.

The images in [41] were enhanced using four techniques: rotation, scaling, horizontal flipping, and addition of Gaussian noise. As a result, the training set was expanded to five times the size of the initial training set.

Furthermore, other studies have applied augmentation to the same dataset more than once in order to expand it and increase model accuracy. As an illustration, the number of training images was increased in [42] using augmentation techniques such as flipping, rotating, and skewing in two stages, first dataset preparation and then data preprocessing. The results showed that the increase in accuracy was both efficient and consistent.

#### 7.1.2. Photometric Transformations

Photometric transformations are used less frequently than the methods described above. They include blurring, sharpening, and adjusting the brightness and contrast. Images can be enhanced using these augmentation techniques to create a generalized model by incorporating potential image variability caused by various imaging conditions [72]. Various random photometric transformations with random parameters, such as sharpening, Gaussian blur, brightness, and contrast adjustment, have been used. In [68,73,74], data augmentation techniques were used to avoid model overfitting, with different variants of the source images generated by applying random photometric transformations such as blurring, sharpening, and contrast adjustment. It can be observed that previous studies sometimes combine the deployment of geometric transformation methods with photometric transformations methods. For example, in [53], the authors used data augmentation techniques to increase the number of training samples and improve generalizability. The augmentation process they used included cropping, probability blur, adding a random amount of Gaussian noise, changes in brightness and contrast, and random horizontal flipping. In [75], the authors implemented augmentation techniques such as cropping, blurring, adding a random amount of Gaussian noise, brightness and contrast changes, and random horizontal flipping in order to effectively increase the number of training samples for improved generalizability. However, it was reported that rotation and shearing had a negative impact on performance, and thus these augmentation methods were avoided.

### 7.2. Deep Learning Data Augmentation Approach

The size and diversity of datasets used to train DL models should be increased to better detect COVID-19. Another augmentation technique based on DL techniques that has been implemented by a number of researchers involves Generative Adversarial Networks (GAN). For example, in [76], the authors applied two data augmentation methods based on basic image alterations and GANs to improve COVID-19 detection from X-ray and CT images. Similarly, in [77], a GAN was used to overcome over-fitting problems and to generate more images from a limited X-ray image dataset.

Alternatively, other researchers have applied both traditional and DL augmentation approaches. For example, Chowdhury et al. [68] applied two different augmentation approaches to X-ray images, using both rotation and translation to generate a balanced training set for CNN models. Sedik et al. [76] used a variety of traditional image transformation methods along with the data augmentation capability of GANs to multiply the size of the dataset by ten. Their results showed improvements in detection accuracy, logarithmic loss, and testing time compared to results obtained without the use of augmented data. In a similar vein, Loey et al. [78] recommended combining traditional data augmentations with CGAN to increase the number of CT images and improve classification performance.

Although data augmentation is widely used in detecting COVID-19 from images, it should be noted that there are studies that do not use data augmentation to detect COVID-19, raising debate concerning the use of data augmentation in general. However, none of these studies explain why particular augmentation steps were included or excluded, and most studies do not compare models before and after augmentation (Table 5).

## 8. Segmentation

The term “segmentation” refers to the division of an image into separate sections. Segmentation is considered a second type of computer-aided diagnosis system that involves splitting X-ray and CT scan images into meaningful areas. It delineates the regions of interest (ROI), such as lung, lobes, bronchopulmonary segments, and infected regions [79]. In addition, segmented areas can be utilized to extract features for diagnosis and other applications. In ROI, lung region-oriented methods (i.e., separate total lung and lung lobes from other background regions in a CT or X-ray) are considered a prerequisite step in COVID-19 detection [80,81]. The lesion lung region methods aim to separate the affected region from other lung regions [81,82]. Due to small variations in shape and texture of the lesion region, it is necessary to locate the affected region, which is regarded as a difficult detection challenge [79].

There are two types of segmentation: traditional segmentation and classification segmentation.

### 8.1. Traditional Segmentation

In [37], Local Binary Patterns (LBP), Frequency Decoded LBP (FDLBP), Quaternionic Local Ranking Binary Pattern (QLRBP), Binary Gabor Pattern (BGP), Local Phase Quantization (LPQ), Binarized Statistical Image Features (BSIF), Census Transform Histogram (CENTRIST), and Pyramid Histogram of Oriented Gradients (PHOG) were all considered for comparison purposes. The BSIF with SVM classifier produced a 90.5% accuracy score using the local texture descriptors. The use of temporal and spatial data in two-stage object detection significantly improved the performance of micro-lesion detection.

### 8.2. Deep Learning Segmentation

Deep learning-based AI solutions have been developed to help radiologists with their work and to enhance segmentation accuracy. CT scans produce high-quality 3D images; DL is commonly used to segment ROIs in CT. Although, X-rays are more generally available than CT images, the segmentation of rib projections onto soft tissues in 2D often confuses the image contrast, making X-ray image segmentation more difficult. DL models such as Fully Convolutional Network (FCN), SegNet, U-Net, UNet++, VB-Net, and Res2Net have been used to segment the lung region of X-ray and CT images for COVID-19 detection, and are briefly discussed below.

- The FCN architecture has been employed for lung segmentation in COVID-19 patients. In this architecture, FC layers are replaced with convolutional layers to record the output as a local map. These maps are up-sampled using the previously mentioned method, which employs backward convolution learning with certain stride size. A 1 × 1 convolution layer at the network’s end produces the corresponding pixel label as the output. The output detail quantity of this layer is constrained by the current stride size in the deconvolution stage. Several skip connections have been introduced to the network from the lower levels to the end layer to address this issue and improve the quality of the results [83].
- The SegNet decoder is designed in such a way that an up-sampling layer is positioned in the decoder for each down-sampling layer in the encoding section, unlike the deconvolution layers in FCN networks. These layers are incapable of learning; when the extraction values of the maximum pooling layer are located, and the remaining output cells are set to zero [84].
- While the U-Net network has the same amount of pooling and up-sampling layers as SegNet, it uses trainable deconvolution layers instead. In addition, the up-sampling and down-sampling layers in this network have a matching skip connection [85]. For COVID-19 diagnosis applications, U-Net is a widely utilized technique for segmenting both lung regions generally and affected lung regions [82,86,87].
- The Res2Net module separates feature maps into numerous subsets and processes them through a set of 3 × 3 filters after 1 × 1 convolution. The outputs are combined, then 1 × 1 convolution is applied. The set of this process is residually structured, and it is consequently called the Res2Net module. The scale dimension (the number of feature groups in the Res2Net block) is a parameter included in this module; as the scale increases, the model learns features with larger receptive field sizes. Res2Net can be used in conjunction with current modules such as cardinality dimension, squeeze, and excitation. In addition, it can be easily combined with several other models, such as ResNeXt, ResNet, DLA, and Big Little Net [88].
- UNet++ is made up of an encoder and a decoder that are linked together by a sequence of layered dense convolutional blocks. Prior to fusion, the semantic gap between the encoder and decoder feature maps are bridged. The encoder extracts feature by down-sampling, while the decoder maps feature to the original image by up-sampling and performs pixel classification to achieve the goal of segmentation. Zhou et al. [89] developed UNet++, which is significantly more sophisticated than U-Net, as it inserts a nested convolutional structure between the encoding and decoding paths. Clearly, such a network can increase segmentation performance. Consequently, the training process is more difficult.
- VB-Net is a modified three-dimensional convolutional neural network that integrates V-Net 14 and the bottleneck structure of V-Net 15. VB-Net is divided into two pathways. The first is a contracting path that uses down-sampling and convolution to extract global image features. The second is a broad approach that includes up-sampling and operations to combine fine-grained image data. A bottleneck structure is implemented into VB-Net 15, which makes it much faster than V-Net 14 in terms of speed. A three-layer stack is used in the bottleneck design. The first layer, with a 1 × 1 × 1 kernel, reduces the number of channels and feeds the data for a conventional 3 × 3 × 3 kernel layer processing, then the channels of the feature maps are restored by another 1 × 1 × 1 kernel layer. The three layers utilize 1 × 1 × 1, 3 × 3 × 3, and 1 × 1 × 1 convolution kernels. The model size and inference time are significantly decreased by combining and minimizing the feature map channels and cross-channel features, which are efficiently fused by convolution. As a result, VB-Net is more suitable for handling huge amounts of 3D volumetric data than the classic V-Net.

Many papers have considered segmentation as a crucial step in diagnosing COVID-19 from medical images. However, there are currently only a few segmentation studies that are directly related to COVID-19. In this study, DL segmentation strategies for COVID-19 detection from X-ray and CT images are summarized.

Arias-Garzón et al. [38] utilized three X-ray image datasets, namely, the Montgomery dataset (138 images), JSTR dataset (240 images), and NIH dataset (100 images), to train U-Net models for segmentation. Despite the seeming lack of data, the volume and variety of images were sufficient to generate a useful segmentation model. For evaluating segmentation tasks, the Dice coefficient and Interception Over Union (IoU) measures showed 0.96% and 94%, respectively. For the detection of COVID-19, the VGG19 classification model was trained using transfer learning, and the results showed an accuracy of 97%. Zheng et al. [86] proposed a weakly supervised 3D Deep Convolutional Neural Network for recognizing COVID-19. U-Net was used to segment the lung areas in each CT volume. The DL algorithm obtained an accuracy of 90%. Wang et al. [90] proposed a lesion segmentation method combining a Deep Supervised Classification Network (DeCoNet) and unsupervised connected component activation regions. In [91], VB-Net was proposed for segmenting and quantifying lesion regions in CT images, which is necessary in order to evaluate disease development and examine COVID-19 longitude. A Dice similarity coefficient of 91.6% was obtained using the suggested technique. Chen et al. [92] used UNet++ to segment the appearance of impacted regions, achieving a per-patient accuracy of 95.24%.

The authors of [68] provided a CT scan-based classification segmentation technique for COVID-19 screening. Subsequently, the application of DL approaches without transfer learning were introduced to tackle the problem of deficient and imbalanced quantity of CXR images in the dataset. In [93], the NABLA-N network was used to segment regions affected by the virus from CT and X-ray images. Rajinikanth et al. [94] suggested an image processing approach for identifying COVID-19 lesions from CT images of the lungs. Initially, the firefly method and Shannon entropy-based multi threshold were used to improve the detection of pneumonia lesions, with Markov random field segmentation then used to identify COVID-19 lesions.

## 9. Feature Extraction

One of the most critical steps in learning rich and informative representations from raw input data to produce accurate and reliable outcomes is the ensuring of effective feature extraction [95,96]. During the feature extraction phase, various features are determined and then extracted to support the learning process of ML and DL models [76]. Each image can generate additional features containing useful information to aid in the classification stage [76]. According to the literature review, authors have adopted both traditional and DL techniques for extracting features.

### 9.1. Traditional Feature Extraction Method

In the context of traditional image processing methods, in [97] the authors applied mathematical morphological approaches to refine and extract the acceptable contours for chest region extraction. Ozturk et al. [98] used four types of feature extraction methods: the Grey Level Co-occurrence Matrix (GLCM), local binary GLCM, GL run-length matrix, and fractal-based texture analysis. Furthermore, the Grey Level Co-occurrence Matrix (GLCM), Local Directional Pattern (LDP), Grey Level Run Length Matrix (GLRLM), Grey Level Size Zone Matrix (GLSZM), and Discrete Wavelet Transform (DWT) algorithms were deployed by Barstugan et al. [99], then classified using a Support Vector Machine (SVM). Tuncer et al. [100] developed the Residual Exemplar Local Binary Pattern (ResExLBP) feature extraction approach with Iterative Relief (IRF) feature selection to detect COVID-19. In [72], eight first-order statistical features (FOSF), 88 grey level co-occurrence matrix (GLCM) features, and 8100 histogram of oriented gradients (HOG) features were employed. Each CXR image yielded a total of 8196 features (8 FOSF, 88 GLCM, and 8100 HOG). The FOSF approach uses the mean, variance, roughness, smoothness, kurtosis, energy, and entropy, among others, to describe the entire image. It is able to easily measure global texture patterns, although it does not consider local neighborhood data. The GLCM and HOG feature descriptors can be utilized to conduct an in-depth texture analysis to solve this issue. The GLCM feature describes the spatial correlation between pixel intensities in radiographic texture patterns based on four unique directions (i.e., 0, 45, 90, 135 degrees), whereas the HOG feature stores local shape/texture information.

### 9.2. Deep Learning Feature Extraction Based on Transfer Learning

**Figure 2:**
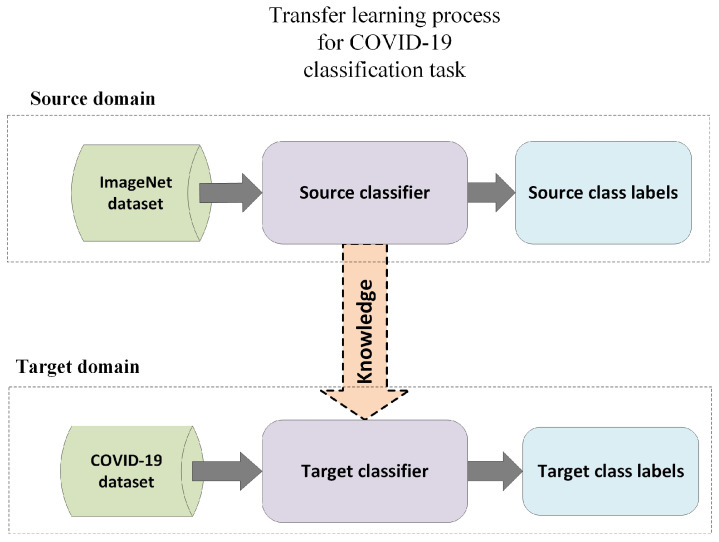
Transfer Learning process.

Transfer learning is the process of transferring knowledge from one context to another in order to enhance the generalization of a new context [101]. It aims to tackle difficult issues for which there are insufficient data or the data labeling technique for supervised learning is expensive. The goal of transfer learning is to make use of information gained by studying models that have been trained using huge datasets. The knowledge acquired from these models is transformed into a set of features and weights that can be exploited by subsequent models with specific goals. Learned low-level features such as edges, shapes, corners, and intensity can be shared throughout tasks, enabling the transfer of information between tasks. In contrast to single-task models, which require similar domains with the same distribution, transfer learning can be implemented in situations in which the domains are different. Transfer learning can be implemented in situations involving two comparable domains with unique tasks, or for similar tasks with different domains [102]. In these situations, single-task models fail due to problems with generalization and over-fitting associated with dataset training. In the context of COVID-19 feature extraction, inductive transfer learning can be utilized to discover and infer a mapping function between image representation and class labels to learn significant features. This necessitates a thorough understanding of information pertinent to the source domain in order to produce rules and assumptions to appropriately represent the domain distribution. This set of assumptions gained from a specific source task in a specific domain can be applied to a target task in a different domain, as shown in Figure 2.

**Figure 3:**
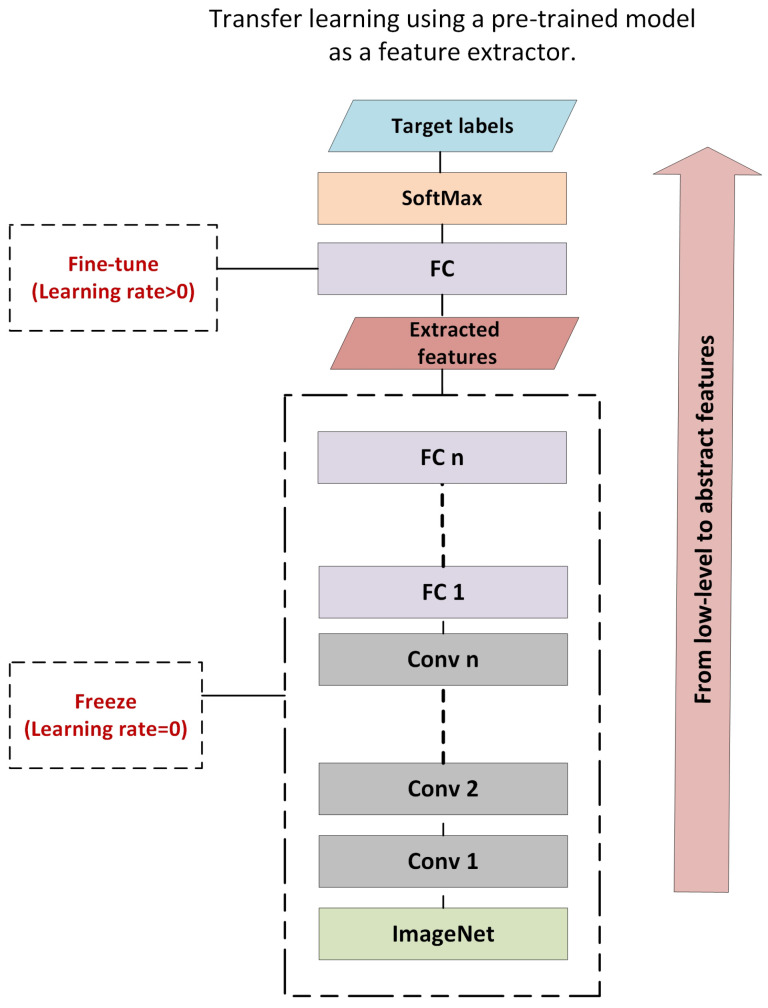
Feature Extraction using Transfer Learning.

Pre-trained models are a collection of models that have been trained on the ImageNet dataset, which comprises around one million images, in order to classify images into one thousand categories. Each layer of these hierarchical designs is intended to learn different types of features that can be extracted from any layer. Transfer learning can be utilized for feature extraction by freezing all the hidden layers and removing the last dense layer that is allocated for classification, as presented in Figure 3.

Most of the research on COVID-19 detection has centered on utilizing pre-trained models for feature extraction and performing extensive comparative studies between different pre-trained model types. To perform feature extraction, this branch of study focuses on three types of images: X-rays, CT scans, and ultrasound images. As opposed to CT scans and ultrasound, X-ray images are the method of choice for COVID-19 identification in most existing studies [34,41,53,65,103]. Valid et al. [53] utilized VGG19 CNN on a pre-trained model as a feature extractor using X-ray images to classify COVID-19 images. Their model achieved 95% accuracy. Basu et al. [103] applied AlexNet, VGGNet, and ResNet as feature extractors to classify X-ray images into normal, pneumonia, other disease, and COVID-19. They found that VGGNet achieved the best results, with 90.13% overall accuracy, with accuracy of 82.98% ± 0.02 and 85.98% ± 0.07 for AlexNet and ResNet, respectively. Ahsan et al. [34] applied feature fusion using histogram-oriented gradient (HOG) and CNN (VGGNet) using fine-tuning to classify COVID-19 X-ray images into COVID-19 versus non-COVID-19. Nayak et al. [41] studied the effectiveness of eight pre-trained models as feature extractors, with only the final FC layer being retained. The models included AlexNet, VGG16, GoogleNet, MobileNet-V2, SqueezeNet, ResNet-34, ResNet-50, and Inception V3. Based on their findings, the best results were obtained by ResNet-34, with an overall accuracy of 98.33%. Jain et al. [65] compared the performance of Inception V3, Xception, and ResNeXt as feature extractors for the classification of X-ray images into COVID-19, normal, and pneumonia. The Xception model provided the highest accuracy at 97.97%. Brunese et al. [104] applied VGG16 as a feature extractor to classify X-ray images as COVID-19 or non-COVID-19, achieving 97% accuracy. It can be observed that VGG19 is commonly used as a pre-trained model for COVID-19 X-ray feature extraction to address the COVID-19 classification issue. Other studies that utilized CT images include [56,74,105,106,107,108,109]. Ardakani et al. [56] performed a competitive study to investigate the effectiveness of a set of pre-trained models as feature extractors. These pre-trained models included AlexNet, VGG16, VGG19, SqueezeNet, GoogleNet, MobileNet-V2, ResNet-18, ResNet-50, ResNet-101, and Xception. Among all networks, the best results were achieved by ResNet-101 and Xception. ResNet-101 achieved an AUC of 0.99, sensitivity of 100%, specificity of 99.02%, and accuracy of 99.51%, while Xception achieved an AUC of 99.4%, sensitivity of 98.04%, specificity of 100%, and accuracy of 99.02%. Zhou et al. [105] applied transfer learning using three pre-trained models, namely, AlexNet, GoogleNet, and ResNet, as feature extraction methods. In addition, they applied ensemble learning using the EDL-COVID classifier to improve the classification results. The proposed models achieved overall accuracies of 98.16%, 98.2%, and 98.56%, respectively. Meanwhile, when using ensemble EDL-COVID, the model achieved 99.05% accuracy. He et al. [107] created a self-transfer learning model for classifying CT-scan images as either COVID-19 or normal. The proposed model used contrastive self-supervised learning in conjunction with transfer learning to discover robust and unbiased feature representations in order to reduce overfitting. The proposed model obtained an F1-score of 85% and an AUC of 94%. Ko et al. [108] applied transfer learning to construct a fast-track COVID-19 (FCONet) network for classifying CT scans as COVID, pneumonia, or non-pneumonia disease. The model incorporated four cutting-edge pre-trained DL models, namely, VGG16, ResNet-50, Inception-v3, and Xception. ResNet-50 outperformed the other three models, with an overall accuracy of 99.87%. Serte et al. [109] developed a COVID-19 classification model based on ResNet-50 and majority voting. The proposed model was then compared to various DL models and fusion techniques. Their results indicated that the ResNet-50 model combined with majority voting beat all other models and fusing procedures, with an AUC of 90% and overall accuracy of 96%. Below, Table 6 presents a summary of the state-of-the-art with respect to pre-trained models.

## 10. Discussion and Future Research Directions

COVID-19 is a new pandemic caused by a novel coronavirus. The World Health Organization (WHO) has classified COVID-19 as a viral outbreak with an extremely high danger of harming millions of lives globally, particularly those with poorer health systems. Early COVID-19 detection is extremely crucial to prevent patients’ the condition from worsening. Therefore, DL algorithms are trained to recognize and categorize lung images for early detection and spread prevention. The COVID-19 diagnostic system is built in stages, beginning with image acquisition and progressing through preprocessing, augmentation, segmentation, feature extraction, and classification. Accordingly, effective feature extraction is one of the most important phases in learning rich and informative representations from raw input data in order to deliver accurate and reliable results. Many of the features described in the literature have been handcrafted by humans with the specific goal of addressing problems such as complex backgrounds, scale differences, and illumination. Unlike deep learning-based features, which are learned from the data, handcrafted features are produced in advance by human experts to extract a predetermined set of features. However, the key issue with handcrafted features is that they are bound to human-defined rules that necessitate domain-specific expertise. In addition, the low-level nature of these types of features limits their applicability to more variedf datasets and classification tasks. Moreover, handcrafted features are computationally expensive due to their high dimensionality, especially with big data. Generally, the design of handcrafted features requires that an optimal balance between accuracy and computing efficiency be achieved. In terms of COVID-19 classification, texture, edge contour, statistical, and color are the most extracted features [118]. More advanced hand-crafted feature extraction techniques include histogram-oriented gradient (HOG), invariant feature transform (SIFT), and bag of words (BoW). On the other hand, deep learning-based features are high-level features learned from image data using complex operations such as convolutional operations. CNN is considered the state-of-the-art feature extraction method for image classification at both the pixel level and image level. It is characterized by its excellent performance and ability to extract hidden and complex patterns without the use of a traditional image processing pipeline [119]. CNN layers serve as a set of feature extractors that are relatively generic and independent of single classification tasks. This is because deep learning acquires a set of features that are directly learned from input images [120]. This facilitates the identification of several levels of representation that can aid in semantic representation by using higher-level features to enhance robustness and generalization. Nevertheless, one of the downsides of CNN-based feature extraction is that it requires the selection of massive training sets, which necessitates human effort and substantial processing power. This is because the lower layers of a CNN extract features that are highly dependent on the input images. Multiple forms of deep learning-based features, including end-to-end CNNs and pre-trained models, have been used to classify COVID-19. For future work, we recommend the development of more end-to-end CNN models and the utilization of feature fusion based on several pre-trained models, as well as on end-to-end models, to generate more generic features and enhance classification accuracy. In addition, handcrafted and automated features can be combined using deep learning.

Preprocessing is another necessary step, helping to restrict the search for anomalies in the background that could affect outcomes [121]. It can be used for image normalization and non-uniform intensity correction to eliminate artifacts and improve the accuracy of the subsequent processes. However, in COVID-19 detection utilizing chest images, preprocessing procedures are not emphasized. Therefore, the classification stage employing a DL algorithm has received the most attention. With respect to this phase, future recommended work is as follows:

- Determining how to automatically choose the best parameters for the preprocessing methods discussed in the literature (resizing, rescaling, normalization).
- Evaluating the effectiveness of COVID-19 detection systems using various preprocessing techniques.

Finally, data augmentation is widely used to achieve consistent results due to the limited availability of medical image datasets for use as training samples in the detection of COVID-19 from images [65]. However, there are studies that do not use data augmentation for COVID-19 detection. In addition, none of the reviewed studies explain why particular augmentation steps were included or excluded, and most studies do not compare models before and after augmentation. Therefore, future works on this phase should focus on discovering the best augmentation approaches discussed in the literature, as well as the best technical combination of these approaches. At present, the efficacy of COVID-19 detection systems is being evaluated using several augmentation approaches.

## 11. Conclusions

The rapid outbreak of the COVID-19 pandemic in December 2019 has led to alarm all over the world. Thousands of illnesses and hundreds of deaths have been reported in practically every part of the world. One of the most crucial diagnostic techniques for classifying and diagnosing infections in humans is RT-PCR. Additional diagnostic methods for diagnosing COVID-19 include X-ray images and CT scans. AI can be utilized for population screening, alarms, infection control advice, learning-prediction models, enhanced drug development, treatment planning, and detailing follow-up for COVID-19 patients. The COVID-19 diagnosis system is being developed through the preprocessing, augmentation, picture segmentation, feature extraction, and classification phases. A thorough analysis of the literature reveals several attempts to develop taxonomies for COVID-19 detection using image processing techniques. Most of these employ categorization criteria based exclusively on classification techniques that are often focused on small or otherwise restricted images. Thus, our review proposes a novel taxonomy for early-stage COVID-19 detection which aims to provide a comprehensive understanding of image processing procedures in the COVID-19 diagnostic context, with consideration of all phases required prior to classification.

## Figures and Tables

**Table 1:** COVID-19 X-ray Image Datasets.

Datasets	Description	Source	References
COVID-19, SARS, MERS X-ray Images Dataset	Includes 423 COVID-19, 134 SARS, and 144 MERS images with the corresponding lung masks	Developed by researchers from Qatar University and the University of Dhaka	Yazan Qiblawey (2022) Last updated: 12 January 2022 http://doi.org/10.34740/kaggle/dsv/3034344
COVID-19 Pneumonia-Normal Chest X-ray Images	Includes COVID-19, normal, and pneumonia images	Collected from different sources including GitHub, Radiopaedia, The Cancer Imaging Archive (TCIA), and the Italian Society of Radiology (SIRM)	Sachin Kumar (2022) Last updated: 14 June 2022 http://doi.org/10.17632/dvntn9yhd2.1
COVID-19 Digital X-rays Forgery Dataset	Includes COVID-19, CM COVID-19, S COVID-19, Normal images, CM Normal, S Normal, Viral Pneumonia, S Viral Pneumonia, and CM Viral Pneumonia	Modified dataset from “COVID-19 Radiography Database”	Nour Eldeen Khalifa (2022) Last updated: 17 March 2022 http://doi.org/10.17632/3bzv6t24ts.1
QaTa-COV19 Dataset	Contains two datasets: the QaTa-COV19 Dataset (Extended) includes 9258 COVID-19 chest X-ray images, while the Early-QaTa-COV19 Dataset includes 1065 chest X-rays	Developed by researchers from Qatar university and Tampere university	aysendegerli (2022) Last updated: 22 February 2022 https://www.kaggle.com/aysendegerli/qatacov19-dataset
Chest X-ray Dataset for Respiratory Disease Classification	Includes five classes from 32,687 chest X-ray radiography images with reasonable resolution (COVID-19, pneumonia, tuberculosis, lung opacity, and normal)	Combination of multiple different datasets gathered from diverse sources	Harvard Dataverse (2022) Last updated: 10 February 2022 http://doi.org/10.7910/DVN/WNQ3GI
COVID Pneumonia dataset	Includes 1950 X-ray images with three classes (COVID, normal, and pneumonia)	Italian Society of Medical, Radiopaedia, and NIH Clinical Center	Redwanul Islam (2022) Last updated: 3 January 2022 https://www.kaggle.com/redwan1010/covid-pneumonia-dataset
xray-binary-covid	Processed COVID-19 X-ray images for DL models. Includes 2000 COVID and 2000 normal images	Information is not available	Aravind Lade (2022) Last updated: 8 February 2022 https://www.kaggle.com/aravindlade/xray-binary-covid
COVID-19 Chest X-ray Image Repository	Includes 900 images. Several of the images are of children or early-stage patients for whom the radiologist noticed no unique imaging findings	Gathered from a variety of online sources	Arman Haghanifar; Mahdiyar Molahasani Majdabadi; Seokbum Ko (2022) Last updated: 2 February 2022 http://doi.org/10.6084/m9.figshare.12580328.v3
COVID-19 Radiography Database	Includes lung masks and 3616 COVID-19 chest X-ray pictures	Developed by researchers from Qatar University, and the University of Dhaka along with their Pakistani and Malaysian counterparts, and medical practitioners conducted the study	Tawsifur Rahman (2022) Last updated: 19 March 2022 https://www.kaggle.com/tawsifurrahman/COVID19-radiography-database
X-ray Image Dataset For COVID-19 Detection (A)	Includes 392 X-ray images (COVID and normal)	Collected from “COVID-chestxray-dataset” in GitHub and “chest-xray-pneumonia” in kaggle	Mohammed Ali-11 (2022) Last updated: 22 March 2022 https://www.kaggle.com/datasets/mohammedali11/xray-image-dataset-for-covid19-detection-a
Curated COVID-19 Chest X-ray Dataset	Includes 9208 chest x-rays (normal, COVID-19, and pneumonia)	Derived from the “Curated Dataset for COVID-19 Posterior-Anterior Chest Radiography Images (X-rays)”	Francis Jesmar Montalbo (2022) Last updated: 25 March 2022 https://www.kaggle.com/datasets/francismon/curated-covid19-chest-xray-dataset
COVID-19 Pakistani Patients X-ray Image Dataset	Includes 390 COVID-19 and 60 normal chest X-ray Images	Developed by researchers from Edinburgh Napier University UK, HITEC University Taxila, and PNEC Karachi, Pakistan along with their collaborators from Kingdom of Saudi Arabia and in collaboration with medical doctors	Muhammad Shahbaz Khan (2022) Last updated: 21 May 2022 https://www.kaggle.com/datasets/muhammadshahbazkhan/covid19-pakistani-patients-xray-image-dataset

**Table 2:** COVID-19 CT Image Datasets.

Datasets	Description	Source	References
COVID-CTset: A Large COVID-19 CT Scans dataset	Includes 63,849 CT images of 377 patients (15,589 obtained from 95 COVID-19 patients and 48,260 CT scan from 282 normal individuals). One of the largest COVID-19 CT scan datasets for AI researchers	Iran’s Negin medical center, located in the city of Sari	Mohammad Rahimzadeh (2022) Last updated: 7 March 2022 https://www.kaggle.com/mohammadrahimzadeh/covidctset-a-large-covid19-ct-scans-dataset
HRCTv1-COVID-19	Includes 181,106 images obtained from 395 patients: GGO (288 cases), Crazy Paving (57 cases), and Air Space Consolidation (27 cases), as well as 23 cases with a negative diagnosis	Sfahan University of Technology, Arak University of Medical Sciences, Isfahan University of Medical Sciences, Islamic Azad University Science and Research Branch	Iraj abedi (2022) Last updated: 5 May 2022 http://doi.org/10.17632/nc5g3zs7g7.2
COVID-19 CT Dataset	Includes 368 medical findings in Chinese and 1104 chest CT scans	Constructed by Shenzhen Research Institute of Big Data (SRIBD), Future Network of Intelligence Institute (FNii) and CUHKSZ-JD Joint AI Lab	Chinese University of Hongkong, Shenzhen, China (2022) https://paperswithcode.com/dataset/covid-dataset accessed on 7 December 2022
COVID-19 Omicron and Delta Variant Lung CT Scans	Includes 14,482 CT scans (12,231 positive for COVID-19 and 2251 negative); data are available as 512 × 512 px JPG images	Collected from patients in radiology centers of teaching hospitals of Tehran, Iran	M Amir Eshraghi (2022) Last updated: 7 February 2022 https://www.kaggle.com/mohammadamireshraghi/covid19-omicron-and-delta-variant-ct-scan-dataset

**Table 3:** COVID-19 Ultrasound Image Datasets.

Datasets	Description	Source	References
Data from: Use of lung ultrasound in neonates during the COVID-19 pandemic	Includes 27 ultrasound images of the lungs of newborns with a suspected or confirmed diagnosis of COVID-19, differentiating between disease-related and non-disease-related alterations	-	Marcia Wang Matsuoka (2021) Last updated: 25 March 2021 http://doi.org/10.6084/m9.figshare.14278767.v1
COVID-19 Dataset	Includes ultrasound images grouped as COVID, pneumonia, and regular	Kafrelsheikh University	Ahmed sedik (2020) Last updated: 9 May 2022 http://doi.org/10.17632/6rs5mnvktk.1

**Table 4:** Comparison of existing related survey papers.

References	Preliminary Stages Before the Detection Process				Database Description
	Preprocessing	Augmentations	Segmentation	Feature Extraction	
[6]	no	no	yes	no	Brief (low)
[14]	no	no	yes	no	Medium
[26]	no	no	no	no	Detail (high)
[29]	no	no	yes	no	Medium
[31]	no	no	yes	no	Brief (low)
[27]	no	no	no	no	Medium
[30]	no	yes	yes	no	Detail (high)
[28]	no	no	no	no	Detail (high)
[33]	no	no	yes	no	Detail (high)
[32]	yes	yes	yes	no	Brief (low)
Our Study	yes	yes	yes	yes	Detail (high)

**Table 5:** Summary of augmentation methods.

Augmentation Methods	Purpose	Augmentation Techniques	Dataset	Author
Geometric Transformations	Reduce the bias caused by the properties of CXR images	flipping, zooming, shifting	CXR images	[67]
	Increase dataset size	rotating, scaling	CXR images	[66]
	Propose a robust technique for automatic detection of COVID-19 pneumonia	rotating, scaling, translation	X-ray images	[68]
	Increase dataset size to achieve efficient and consistent accuracy	flipping, rotating, skewing	X-ray and CT images	[42]
	Solve overfitting problem	rotating, shearing, translation, novel data augmentation	CT images	[69]
	Improve CNN model training and classification accuracy	flipping, rotating, translation	X-ray images	[70]
	Generate more samples	flipping, rotating, translation	X-ray images	[71]
	Prevent overfitting	rotating, zooming, shearing.	X-ray images	[65]
	Increase training set size	flipping, rotating, scaling, Gaussian noise addition	X-ray images	[41]
Photometric Transformations	Enhance images	sharpening, blurring, brightness, contrast adjustment	X-ray images	[72]
	Avoid model overfitting	blurring, sharpening, contrast adjustment	CT images	[73,74]
	Avoid model overfitting	blurring, sharpening, contrast adjustment	X-ray images	[68]
Geometric and Photometric Transformations	Increase training samples and improve generalization	cropping, blurring, Gaussian noise addition, brightness and contrast adjustment, flipping	CXR images	[53]
	Increase training samples and improved generalization	cropping, blurring, Gaussian noise addition, brightness and contrast adjustment, flipping	CT images	[75]
DL Augmentation	Improve COVID-19 detection	Augmentation based on basic image alteration and GANs	X-ray and CT images	[76]
	Overcome overfitting problem and generate more images	GAN	X-ray images	[77]
Traditional and DL Augmentation	Generate a balanced training set	Rotation and translation (CNNs)	X-ray images	[68]
	Assess data augmentation impact on the accuracy of COVID-19 detection	Variety of traditional image transformations and GANs	X-ray and CT images	[76]
	Generate additional images and improve classification performance.	Traditional data augmentations with CGAN	CT images	[78]

**Table 6:** Summary of the state-of-the-art of pre-trained models.

Pre-Trained CNN Models	X-rays Studies	CT-Scans Studies	Advantages	Disadvantages
VGG-family [110]	[34,41,53,103,104]	[41,108]	Allows non-linearity through implementation of small kernels.	The vanishing gradient problem. Slower compared to other models.
ResNet-family [111,112]	[41,54,103]	[56,89,108,109]	ResNets are deeper than VGGs, but “skip connections” make them faster. Avoids the problem of vanishing gradients.	Increased overhead due to: Batch normalization layers implementation. “Skip connections” involve managing levels’ dimensions.
Inception- family [113,114]	[41,54]	[56,89,108]	Inception uses 1 × 1 convolution to minimize dimensions in the deep CNN before using 3 × 3 and 5 × 5 convolutions. Utilize various convolutional filter sizes to extract features at various scales. Appropriate for devices with limited computational capability.	Appropriate for devices with limited computational capability. Some versions of Inception, such as V1, are susceptible to information loss due to the use of relatively large filters, such as 5 × 5 filters, which reduces the input dimensions by a significant margin.
AlexNet [115]	[41,103]	[56,89]	The first CNN model that utilize GPUs for training. Deep architecture allows to learn significant features compared to LeNet. Increased information retention by utilizing the ReLU activation function.	This shallower model struggles to learn image features. In comparison to other models, it takes longer to acquire greater accuracy.
MobileNet [116]	[41]	[56]	Lightweight deep neural network. Small in size, low-latency, and less parameters.	Designed for specific applications such as mobile and embedded vision applications. Focus on light computing at the expense of accuracy. Less accurate than other state-of-the-art networks.
SqueezeNet [117]	[41]	[56]	Smaller and faster compared to other models. Requires less parameters. Requires less bandwidth. Efficient for distributed training. More suitable to on-chip Field Programmable Gate Arrays (FPGA) implementations.	Similar to a fully connected layer with 1 × 1 filters; therefore, it is incapable of spatial abstraction. Squeezing might hinder the flow of information.

## Data Availability

Not applicable.

## Abbreviations

The following abbreviations are used in this manuscript:

MDPI	Multidisciplinary Digital Publishing Institute
DOAJ	Directory of open access journals
TLA	Three letter acronym
LD	Linear dichroism
